# Methylphenidate and Its Impact on Redox Balance and Behavior

**DOI:** 10.3390/jox15050157

**Published:** 2025-09-30

**Authors:** George Jîtcă, Ingrid Evelin Mehelean, Ana Natalia Maier, Carmen-Maria Jîtcă

**Affiliations:** 1 Department of Pharmacology and Clinical Pharmacy, Faculty of Pharmacy, George Emil Palade University of Medicine, Pharmacy, Science and Technology of Târgu Mureș, 540139 Târgu Mureș, Romania; 2 Faculty of Pharmacy, George Emil Palade University of Medicine, Pharmacy, Science and Technology of Târgu Mureș, 540139 Târgu Mureș, Romania; inghridmehelean@gmail.com (I.E.M.); ana.natalia.maier@gmail.com (A.N.M.); 3 Doctoral School of Medicine and Pharmacy, Institution Organizing Doctor’s Degree University Studies, George Emil Palade University of Medicine, Pharmacy, Science and Technology of Târgu Mureș, 540139 Târgu Mureș, Romania; carmenrusz20@gmail.com

**Keywords:** methylphenidate, oxidative stress, behavior, dopamine, adverse reaction

## Abstract

Methylphenidate (MPH) and its active enantiomer, dexmethylphenidate, are widely prescribed as first-line therapies for attention deficit hyperactivity disorder (ADHD), yet their increasing non-medical use highlights significant clinical and toxicological challenges. MPH blocks dopamine (DAT) and norepinephrine (NET) transporters, thereby elevating synaptic catecholamine levels. While this underpins therapeutic efficacy, prolonged or abusive exposure has been associated with mitochondrial impairment, disrupted bioenergetics, and excessive reactive oxygen species (ROS) production, which collectively contribute to neuronal stress and long-term neurotoxicity. Growing evidence suggests that the gut–brain axis may critically influence MPH outcomes: diet-induced shifts in microbiome composition appear to regulate oxidative stress, neuroinflammation, and drug metabolism, opening potential avenues for dietary or probiotic interventions. From a forensic perspective, the detection and monitoring of MPH misuse require advanced methodologies, including enantioselective LC–MS/MS and analysis of alternative matrices such as hair or oral fluids, which enable retrospective exposure assessment and improves abuse surveillance. Despite its established therapeutic profile, MPH remains a compound with a narrow balance between clinical benefit and toxicological risk. Future directions should prioritize longitudinal human studies, biomarker identification for abuse monitoring, and the development of mitochondria-targeted therapies to minimize adverse outcomes and enhance safety in long-term treatment.

## 1. Introduction

Methylphenidate (MPH) and its D-isomer, dexmethylphenidate, represent the first-line treatment for attention deficit hyperactivity disorder (ADHD). It is one of the most commonly prescribed psychostimulants, primarily acting through a dopaminergic mechanism by inhibiting dopamine (DA) reuptake, and to a lesser extent that of norepinephrine (NA) and serotonin (5-HT). Because of this, concerns have been raised about its potential for abuse among young people, which is why the substance is dispensed only with a special medical prescription. MPH can be considered an alternative to amphetamines and is widely used by students due to its potential to enhance concentration, with these effects being the main reason for its use in the first place [1], but due to its mechanism of action, the purpose of its use has shifted toward other goals [2]. Thus, in a study published in 2018, it was reported that 1.9% of the U.S. population aged 12 and older had used prescription amphetamines or MPH for non-medical purposes [3].

**Figure 1:**
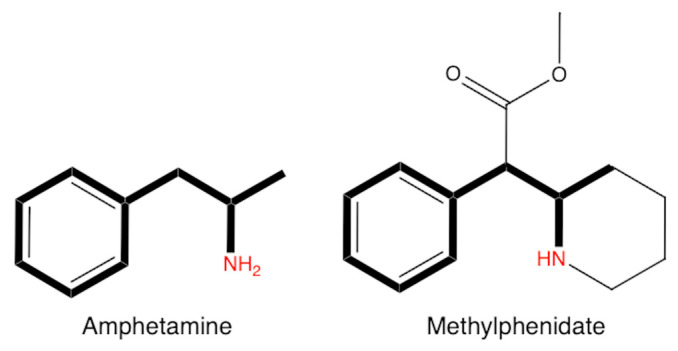
Chemical structure of amphetamine and methylphenidate.

Before discussing the mechanism of action, it is important to consider the influence of chemical structure on pharmacological effects. Both compounds are derivatives of phenethylamine, containing a phenyl ring and an ethylamine group, as seen in Figure 1. The presence of these two functional groups enables interaction with monoamine transporters. In the case of amphetamine, a hydrogen atom on the α-carbon is replaced with a methyl group, whereas MPH has a more complex chemical structure. MPH is a piperidine derivative, with a piperidine ring attached to the phenyl ring. Due to this structural difference, the substances interact differently with transporters: MPH primarily inhibits reuptake proteins, while amphetamines can both inhibit and use these transporters to promote monoamine release into the synaptic cleft [4,5].

**Figure 2:**
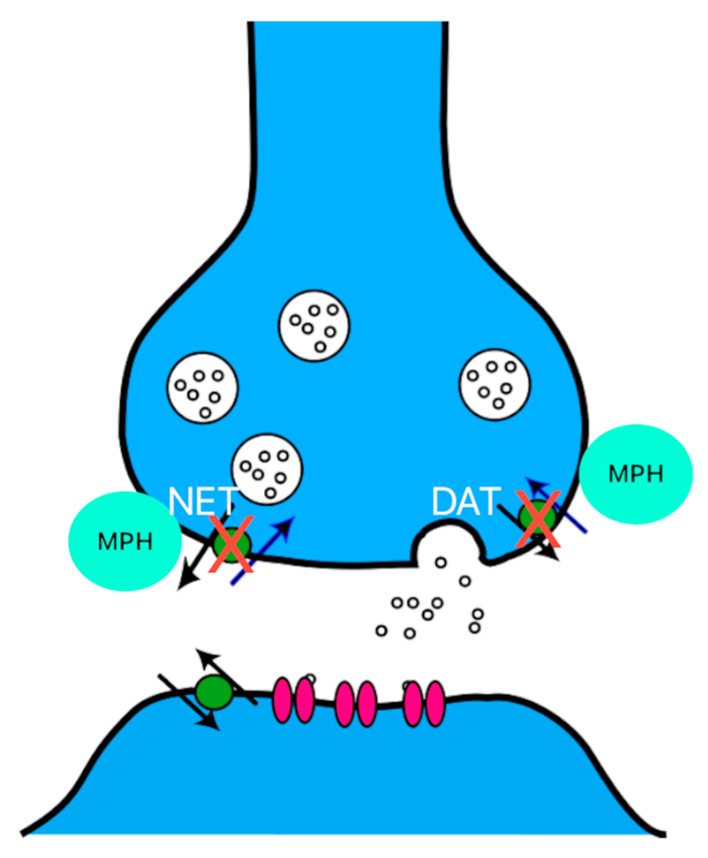
Methylphenidate (MPH) blocks the dopamine transporter (DAT) and the norepinephrine transporter (NET), leading to elevated concentrations of dopamine and norepinephrine in the synaptic cleft. The therapeutic effect is mainly due to the blockade of DAT.

The mechanism by which DA and NA levels increase is primarily related to the blockade of reuptake pathways, similarly to cocaine. Specifically, the DA transporter (DAT) and the norepinephrine transporter (NET) are inhibited, leading to elevated concentrations of DA and NA in the synaptic cleft, as seen in Figure 2. However, the mechanism of action of MPH differs from that of amphetamines in one key aspect. While MPH inhibits monoamine reuptake, it does not promote the release of these amines, unlike amphetamines, which facilitate their release via the vesicular monoamine transporter 2 (VMAT2) [6,7]. In addition, amphetamines involve several other mechanisms of action. Some studies also suggest that the therapeutic effects are primarily mediated by the stimulation of D1 receptors, whereas the rewarding effects are associated with the activation of D2 receptors [6,8,9].

## 2. Metabolism Influence

MPH is predominantly metabolized in the liver through enzymatic hydrolysis catalyzed by carboxylesterase 1 (CES1), resulting in the main inactive metabolite, ritalinic acid. CES1 is an enzyme primarily expressed in the liver, and its genetic variations can significantly influence plasma levels and therapeutic response. Unlike many other psychostimulant drugs, MPH involves minimal participation of the CYP450 enzymatic system, which reduces the potential for drug interactions via this pathway [10]. Thus, the possibility of using MPH in combination with other substances that can alter the pharmacokinetics and metabolism of this drug should be considered, with the aim of increasing the duration and intensity of its effects (aripiprazole, perphenazine, thioridazine, fluoxetine). Alcohol is one such substance with a strong inhibitory effect on CES1 [11,12]. Thus, when MPH is combined with alcohol, antidepressants, or other drugs, the risk of oxidative stress is increased. The simultaneous use of MPH and alcohol is among the most notable interactions. In such cases, a compound called ethylphenidate forms in the liver, and the plasma concentration of d-MPH can increase by up to 40% [13]. In the case of antidepressants, MPH can promote the occurrence of serotonin syndrome [14]. Furthermore, reports accumulated from the concurrent use of alcohol and cocaine or methamphetamine demonstrate an additive effect of reactive oxygen species (ROS), causing oxidative damage to lipids, proteins, and DNA [15].

For ADHD or narcolepsy, MPH is administered orally in controlled doses, but when used for recreational purposes, administration can be intranasal or intravenous and at doses higher than therapeutic ones. These high doses expose users to adverse reactions (psychosis, cardiovascular dysfunctions, dependence). Table 1 presents the therapeutic and abusive doses along with their purposes.

## 3. Methylphenidate and Oxidative Stress

Oxidative stress is a redox imbalance between the production of reactive oxygen species (ROS) and the capacity of endogenous antioxidant systems to neutralize them. Under normal conditions, ROS participate in numerous physiological processes (cell signaling, regulation of gene expression), but when this balance is disturbed, elevated levels of ROS can damage lipids, proteins, and nucleic acids, leading to cellular dysfunction and, over time, the development of chronic diseases [23,24]. The effects of oxidative stress strongly depend on the intensity and duration of exposure to ROS. At low levels, these reactive species play a protective role by activating adaptive signaling pathways and DNA repair mechanisms. In contrast, moderate and chronic levels can lead to persistent inflammation, epigenetic changes, and mitochondrial dysfunction. At high levels, oxidative stress causes irreversible damage to genetic material, lipid peroxidation of membrane lipids, and activation of apoptotic pathways [24,25,26]. Oxidative stress cannot be measured directly, but it can be assessed indirectly. One such biomarker is 8-hydroxy-2’-deoxyguanosine (8-OHdG), which results from the oxidation of guanine in DNA [27]. Another important process associated with oxidative stress is lipid peroxidation, especially of unsaturated fatty acids in cell membranes. The most studied secondary product of this reaction is malondialdehyde (MDA), which can be measured using the thiobarbituric acid reactive substances (TBARS) method [28]. At the protein level, oxidative stress causes structural modifications through amino acid carbonylation, an irreversible process that alters enzymatic function and promotes protein aggregation. Measuring the total content of carbonyl groups is widely used in studies of cellular aging and neurodegenerative diseases [29,30,31]. Additionally, the total antioxidant capacity or the activity of antioxidant enzymes (SOD, CAT, GPx) can also be measured.

The clinical efficacy of MPH is based on its mechanism of action, which involves the inhibition of DAT and NET; however, the same mechanism also contributes to oxidative stress generation when high doses are used illicitly as a stimulant. In the generation of oxidative stress, the accumulation of DA in the synaptic cleft is the main cause. Thus, DA can undergo auto-oxidation or be metabolized by monoamine oxidase type B (MAO-B), generating reactive species (hydrogen peroxide, superoxide anion, hydroxyl radical) [32].

Data from the literature suggest that MPH influences energy balance through mitochondrial complexes. Thus, depending on the mode of administration, dose, and location, the activity of complexes II and IV of the respiratory chain increased [33,34]. Also, depending on the mode of administration and dose, the activity of antioxidant enzymes is altered [35,36]. In another animal study, chronic administration of MPH was observed to induce increased lipid peroxidation and decreased CAT activity [37]. Not only is enzymatic activity altered, but the ratio between reduced glutathione and oxidized glutathione is also affected [38]. This is supported by the fact that by inhibiting DAT, the level of DA in the synaptic cleft increases, it oxidizes and generates hydrogen peroxide, which raises the level of oxidized glutathione [7,39,40,41,42]. Data of few experiments are shown in Table 2.

The overall picture created by the studies included in the table suggests that MPH has an inverted “U”-shaped dose–response curve, meaning that beneficial effects occur at low doses, while exposure to high doses tends to cause harmful effects. This dynamic is best observed in the case of anxiety and memory. In rodents, low doses and short-term exposure can be neutral or even beneficial for cognitive performance, improving, for example, spatial learning in pre-adolescents. In contrast, high doses or chronic administration are associated with hyperactivity, increased sensitivity to stress, and memory problems, sometimes preceded by a short initial period of improvement. This pattern is compatible with the idea that there is an optimal level of DA and NA in the prefrontal cortex. Once this level is exceeded, other brain circuits are activated, which favor anxiety [43,44,45,46]. In terms of anxiety, the apparent contradiction between “anxiolysis” (reduction in anxiety) and “anxiogenesis” (induction of anxiety) is explained by the magnitude of the dose (a moderate stimulation of the prefrontal cortex versus a hyper-stimulation that activates the amygdala), the baseline state of the individual (sex, age, and initial level of anxiety), and the type of test used. This integration shows that the anxiolytic effects of low doses can coexist with the anxiogenic effects of high doses or in situations that test reward/aversion [48,52,64]. In the field of memory, the paradox between “improvement” and “impairment” depends on the type of memory tested, the timing of drug administration (before, during, or after learning), and the duration of exposure. For example, spatial performance in young animals may improve at moderate doses, whereas memory consolidation in recognition tests may be impaired by higher doses or by administration at crucial times for synaptic plasticity. Furthermore, chronic exposure has a different profile than a single dose, with initial benefits followed by a decline, likely due to receptor adaptations [44,45,46,53]. Another important source of inconsistencies is related to developmental stages. The dopaminergic and noradrenergic systems are in the process of remodeling during pre- and peri-adolescence. Therefore, the same doses may have different effects in young people than in adults. Studies in young animals often show persistent behavioral changes (hyperactivity, stress sensitivity), while in adults, the effects seem to be rather reversible. These discrepancies explain why some studies show pro-cognitive effects in early phases and negative effects with prolonged exposures [43,58,59]. In addition, differences in methodology (maze type or memory tasks), gender, and background stress may alter the direction of the effect. Under stress, a dose of MPH that would normally be advantageous may become disadvantageous. Recent literature on neuroinflammation suggests that the state of the immune system may influence the direction of the MPH effect [49,50,61]. Neurochemically, evidence shows that dopaminergic and noradrenergic changes are reversible but functionally relevant, which is consistent with the idea of initial benefits sometimes followed by tolerance [43,55,56,57]. Last but not least, the methodological quality of the studies varies. Many are conducted in rodents with small sample sizes, and differences in locomotor activity are not always adequately controlled, potentially mimicking anxiety-like effects in some tests. Also, the timing of testing relative to dosing is not uniform.

## 4. Use Outside Clinical Indication

Recently, MPH has become the subject of an increasingly widespread phenomenon regarding its non-therapeutic use, especially among students. Outside its specific indications, this medication is mainly used to enhance cognitive performance, increase concentration, and allow for sustained long study periods during exam times. Thus, MPH has entered the category of ‘smart drugs’ which includes substances abused in an attempt to optimize mental functioning. Studies conducted in various countries support the scale of this phenomenon. A study from the USA showed that 6.9% of respondents have used MPH at least once without a medical prescription [65]. More recent studies indicate a high prevalence of use in the last 12 months, ranging between 4% and 10.8%, and between 8% and 35% for lifetime use [66].

Also, in Australia, a study reported a lifetime prevalence of 6.5% and 4.4% for use in the past year [67]. In South Africa, 11% of medical students admitted to recent MPH use, 68% indicated academic performance as the main reason, and 70% obtained it from a doctor [68].

This unauthorized use of MPH is supported by the belief that it improves cognitive abilities. Many who use it do so before exams to increase concentration or to study throughout the night. Sources for obtaining the drug vary and include roommates, friends, or even relatives who have a medical prescription. Some users take it orally, but there are also cases of nasal administration (‘snorting’), which significantly increases medical risks [69].

## 5. Methylphenidate and Adverse Reaction in Relation to Oxidative Stress

Scientific evidence does not clearly support the cognitive benefits of non-therapeutic use. Some studies indicate that students who abuse MPH actually tend to have poorer academic performance than those who do not use it. Additionally, there are numerous adverse effects they are exposed to, including insomnia, anxiety, irritability, hypertension, tachycardia, as well as severe psychiatric disorders such as paranoia, psychosis, or depression. In the long term, there may also be a risk of dependence [70].

At the synaptic level, ROS exert toxic effects on proteins involved in transmission, on receptors, mitochondria, and neuronal plasticity, causing significant disruptions in essential cognitive functions such as learning and memory. Physiological levels of ROS are necessary for synaptic plasticity. Elevated ROS levels generate inflammation, oxidation of synaptic proteins and lipids, and destabilization of synapses [71,72]. Additionally, excessive loading of Ca^2+^ through NMDA receptors causes excitotoxicity, microtubule damage, and mitochondrial fragmentation, which amplifies ROS generation [73]. Furthermore, oxidation of synaptic proteins hinders the release and reuptake of neurotransmitters, leading to inconsistent or absent synaptic transmissions. In an ADHD model treated with MPH, increased lipid peroxidation and decreased activity of antioxidant enzymes were observed, reflecting a profound disruption of the brain’s energy and synaptic function [74].

Administration of MPH is accompanied by the risk of oxidative stress. This adverse reaction does not manifest uniformly among users but is strongly influenced by individual vulnerabilities.

Polymorphisms in certain genes involved in detoxification and cellular protection, such as GSTP1 or GPx1, can significantly reduce the efficiency of antioxidant enzymes [75].

Nutrition is another key factor in defending against oxidative stress. A diet low in antioxidants can leave the body exposed to the harmful effects of MPH, while a diet rich in antioxidants can activate protective cellular pathways. In children and adolescents, metabolism is more active, and the nervous system is still developing, which can make the brain more susceptible to oxidative stress. A study conducted on young rats showed that exposure to MPH led to a significant increase in oxidative stress markers in the prefrontal cortex [76].

The presence of other medical conditions or chronic inflammatory diseases further amplifies the risks. In diabetes, persistent hyperglycemia leads to the formation of advanced glycation end products (AGEs), which stimulate RAGE receptors and increase systemic inflammation and free radical production [77]. Also, in chronic inflammatory diseases, elevated levels of proinflammatory cytokines lead to the activation of NADPH oxidase, increasing the generation of ROS [76].

There is growing concern regarding the role of oxidative stress in the pathogenesis of psychiatric disorders, including major depression, bipolar disorder, schizophrenia, and anxiety, with the brain being vulnerable to redox imbalances. Excessive oxidation of lipids, proteins, and DNA can cause damage between synapses, leading to neuroinflammation and neuronal dysfunction. In patients with major depression, levels of 8-oxo-2′-deoxyguanosine in plasma and urine were elevated, and the level of isoprostane F2 in cerebrospinal fluid was increased, suggesting the presence of oxidative stress [78].

Bipolar disorder is also characterized by a significant redox imbalance. A meta-analysis dedicated to oxidative stress markers identified an increase in lipid peroxidation products and nitric oxide [79], while other studies found no significant changes [80].

Adding to this analysis is the interaction between proinflammatory cytokines and oxidative stress, highlighting the role of TNF-α and IL-6 in disrupting redox mechanisms [81]. In schizophrenia, mitochondrial complex I dysfunction and increased levels of lipid peroxidation products have been demonstrated, suggesting that oxidative stress contributes to cognitive decline and disease progression [80,82].

A review presents clinical and preclinical evidence linking anxiety and autism to redox imbalances, and animal studies confirm the role of oxidation in exacerbating anxiety symptoms [83]. Another study highlighted a causal relationship between oxidative stress and psychiatric disorders [84].

Thus, there is a basis supporting the hypothesis that oxidative stress is a common pathogenic factor in major psychiatric disorders. Antioxidant interventions may represent an important step in the integrated approach to these conditions, so understanding the pathology and effectively treating psychiatric disorders must also include maintaining redox balance. The use of psychoactive substances (amphetamines, cocaine, opioids, alcohol, cannabis) has a profound impact on the brain, not only by altering neurotransmitter release but also by inducing oxidative stress and inflammation, which facilitate the onset of psychoses, anxiety, and depression. Psychostimulant substances are modifiers of redox balance that transform occasional use into addiction.

At the molecular level, the central mechanism of most narcotics involves DA metabolism [7,85]. Moreover, the consumption of psychostimulant substances affects glutamatergic homeostasis and neurotransmitter transport, which reinforces dependence and increases the risk of relapse [86]. In this context, memory, attention, and emotional self-regulation are affected in the long term.

In cases of chronic use, psychoses induced by cocaine or amphetamines are more frequent. These substances excessively stimulate NMDA receptors, causing excitotoxicity and further increase in ROS. Alcohol abuse, in turn, generates chronic inflammation and accumulation of ROS; this primarily affects the prefrontal cortex and hippocampus [87,88,89]. MDMA consumption causes hyperthermia and serotonergic neurotoxicity [90].

At the epigenetic level, studies show that ROS affect enzymes involved in DNA methylation, the dopaminergic system, and neuronal plasticity. This “redox imprinting” on the genome predisposes to increased vulnerability to psychosis, anxiety phenomena, and mood disorders. In rats chronically exposed to methamphetamine, a reduction in glutathione in the nucleus accumbens and prefrontal cortex is observed, along with compulsive and anxious behaviors and cognitive impairment [85].

The use of MPH outside clinical indications opens the way to significant psychiatric disturbances such as psychoses, severe anxiety, and mood changes. These are often facilitated by oxidative stress induced by uncontrolled use. Although usage frequency is low, consumers can develop hallucinations, delusional ideas, or paranoia, especially after sustained occasional use, exacerbated by genetic predispositions or redox deficits. The mechanism behind symptom onset is represented by overstimulation of D2 dopaminergic receptors and neuronal oxidative stress, which destabilizes synaptic homeostasis, favoring excitotoxicity and microglial inflammation. Regarding anxiety and mood disorders, the use of MPH—even at therapeutic doses—is associated with palpitations, increased blood pressure, and difficulty relaxing. Furthermore, symptoms such as severe insomnia, heightened irritability, and mood instability linked to MPH abuse can cause additional redox imbalance. These behavioral disturbances amplify microglial activation and the production of proinflammatory cytokines, increasing susceptibility to depression and anxiety disorders [91,92,93,94].

In the long term, synaptic changes induced by oxidative stress may lead to persistent neurological disorders: decreased neurogenesis in the hippocampus, reduced dendritic density, and mitochondrial dysfunction. These changes are correlated with deficits in memory and attention, as well as chronic emotional disorders. MPH abuse, similar to other psychostimulant substances, affects not only the central nervous system but can also impact other organs (heart, liver, kidneys). This effect is due to sympathetic overstimulation and promotes the generation of ROS.

MPH toxicity, in the context of chronic use, extends beyond the central nervous system, affecting other organs (retina, liver, kidneys, heart). Thus, adverse effects such as cataract, glaucoma or visual loss have been reported, and these have been attributed to oxidative stress, the retina being an organ sensitive to redox disorders. A study on 661 W cell cultures shows that exposure to MPH induces apoptosis by activating caspases 3 and 9. It also increases the level of oxidative stress by decreasing the level of GSH (the reduced form of glutathione) and increases the level of MDA and activates markers of autophagy (LC3B, Beclin-1) [95]. Regarding the activation of autophagy, oxidative stress represents such a factor, suggested in a study conducted by Desideri et al. This study states that the oxidation of GSH induces autophagy in the absence of other triggering factors [96]. Oxidative stress also plays a role in the induction of apoptosis or cell proliferation via JAK/STAT [97]. These data are supported by animal studies. Thus, in a rat study, the retinal structure was not modified, but the number of cone and rod photoreceptors, the number of scotopic photoreceptors, synapsin and PSD95 levels decreased. The same study showed that microglia activation and decreased CX3CR1 receptor expression is correlated with neuroretinal inflammation and increased blood-retinal barrier permeability [98]. These phenomena may also be due to ROS, as the authors observed increased expression of iNOS and some proinflammatory cytokines (IL-6, TNF) [99,100]. It is important to note that these effects may occur with chronic use, while with acute use visual performance is improved [101,102].

The activity of the heart is closely related to the functionality of the kidneys, and the effects of MPH on the heart have begun to be described in the paragraph dedicated to the kidneys. The effects on the heart derive from the mechanism of action of MPH, being a neurosympathomimetic, with the increase in the concentration of catecholamines, thus stimulating the adrenergic nervous system. This mechanism of action can explain the effects on the heart (arrhythmias, myocardial infarction) of MPH, but also of other substances with a similar mechanism of action. These phenomena appear at the beginning of treatment and increase with increasing doses [103]. Heart cells are sensitive to oxidative stress, and myocardial damage has been demonstrated in multiple studies on experimental animals, which were administered MPH [104,105]. In case of myocardial infarction, due to ischemia and reperfusion, the production of ROS increases due to the oxygen supply after reperfusion. This massive increase in ROS exceeds the capacity of endogenous antioxidant systems, leading to oxidative stress, membrane lipid peroxidation, mitochondrial dysfunction, and activation of apoptotic and necrotic pathways, resulting in the extension of myocardial injury and worsening cardiac dysfunction. A case is described in the literature in which a patient complained of chest pain and increased troponin levels following an increase in the dose of MPH. These effects occurred not only as a result of the increase in the dose, but also due to the association with venlafaxine (used for therapeutic purposes), and the alteration of the balance between the adrenergic and cholinergic systems [106]. Thus, the toxic effects of MPH on the heart are mainly due to the mechanism of action through the influence exerted on catecholamines, and less to the direct influence of MPH [107].

A case study of an adolescent who consumed a single dose of MPH described the onset of acute cardiomyopathy, arrhythmias, pericarditis, and elevated cardiac biomarkers (troponin I, CK, CK-MB) [108]. Due to its mechanism of action and excessive stimulation of β1 receptors, apoptosis of cardiomyocytes is triggered [109]. A study on rats showed that usual doses of MPH increased the GSH/GSSG ratio in the prefrontal cortex and hippocampus. At the cardiac level, the GSH/GSSG ratio also remained elevated, but histologically interstitial edema and vascular congestion were observed, while at the renal level, NF-kB was activated [110].

A review including 10 studies with approximately 4.2 million individuals treated with ADHD medications found a moderate increase in the risk of arrhythmias and sudden death (RR ≈ 1.39, confidence interval 1.06–1.83). Specifically for MPH, the risk ratios for sudden death/arrhythmias, stroke, infarction, and death from any cause were 1.46 (1.03, 2.07), 0.92 (0.7, 1.21), 0.97 (0.77, 1.23), and 1.00 (0.49, 2.04), respectively. This study found no direct correlations between medication and causes of death but did not exclude the risk of these events [111]. These findings are supported by another study suggesting that MPH treatment should be individualized [112].

Another organ affected by MPH use is the liver, but this toxicity is cumulative, as under therapeutic conditions, liver toxicity is rarely reported. A case report describes a case in which a 12-year-old boy required liver transplantation after developing severe hepatitis and liver failure. After all causes of hepatotoxicity were excluded, MPH was confirmed to be the substance that caused the toxic reaction [113]. In a study in mice, administration of high doses of MPH (75–100 mg/kg) caused hepatotoxicity, by increasing ALT levels and histology, and the association with β agonists increased toxicity by increasing hepatic MPH concentration [114]. In another study in rats, chronic administration (2 mg/kg) caused an increase in ALT levels [115]. Also, the concomitant use of MPH with alprazolam induces hepatotoxicity, demonstrated by the increase in liver enzymes, but also in oxidative stress [116]. MPH-induced hepatic toxicity occurs especially when combined with other drugs, as MPH is metabolized via the CES1 pathway. In a rat study, chronic administration of MPH together with alprazolam led to increased ALT, AST, alkaline phosphatase, and changes in redox balance [116].

The kidneys are important organs in maintaining the hydro-electrolyte balance and eliminating metabolites generated from other substances, so that they do not accumulate and favor the occurrence of toxicity. These organs are sensitive to the substances and doses used, so that in the case of MPH, chronic or abusive use can affect the kidneys. In a study on rats, the administration of doses of 20 mg/kg, for 21 days induced tubular inflammation, decline in the mean of Bowman’s space thickness and renal corpuscle’s volume, suggesting the activation of nephrotoxicity mechanisms [117]. An animal model using rats given MPH twice daily at a dose of 5 mg/kg showed that the GSH/GSSG ratio in the brain and heart increased (with a decrease in GSSG but no increase in GSH), but in the heart it caused interstitial edema, vascular congestion, and the presence of fibrin-like material in the interstitial space. Necrotic areas appeared in the kidneys, with cellular disorganization, cellular infiltration, and NF-kB activation [110]. These phenomena are supported by another study in which the application of 10 μg/mL of MPH to isolated rat kidneys reduced the glomerular filtration rate, possibly through interaction with vasodilator prostaglandins [118]. In an animal model, the administration of ATP reduced MPH-induced oxidative stress, favoring antioxidant mechanisms and reducing renal toxicity [119].

Renal function is also affected. Histopathological studies reveal tubular necrosis, cellular infiltration, and alterations in Bowman’s space, accompanied by dysfunctions in autophagy, inflammation, and apoptosis. Additionally, redox balance was disrupted, as indicated by increased activity of SOD and CAT enzymes [117]. Table 3 summarizes toxic effects on retina, liver, kidneys, and heart.

Throughout this review, the adverse effects of MPH have been discussed. In this perspective, it should be noted that some studies show the occurrence of neurotoxic effects at very high doses, which are correlated with a considerable reduction in dopaminergic neuronal density in the substantia nigra [122], data that are confirmed by other studies with a decrease in the density of serotonergic and cholinergic neurons [123]. In another study, high doses of MPH caused a reversible increase in DAT and D1 receptors, these changes being reversible [124]. These effects of MPH on the central nervous system have been detailed in previous chapters of this article. However, the positive effects of MPH should not be neglected. These positive effects occur at therapeutic doses and provide long-term benefits, with a decrease in the risk of suicide, criminal behavior or substance abuse. In a prospective study, the administration of MPH in a delayed form reduced alcohol consumption and smoking, suggesting that careful monitoring of MPH treatment brings benefits [125]. These data are consistent with other studies that have concluded that psychostimulant substances used in attention disorders, including MPH, do not increase the risk of abuse of other substances (cocaine, marijuana). However, the data do not confirm that they protect or increase the risk of later use [126].

## 6. Methylphenidate and Mitochondrial Homeostasis

The mitochondrial influence of MPH follows a hormetic relationship, which involves dose and duration dependence. Thus, low doses can be neutral from this point of view or induce adaptive responses [24], while exposure to high doses and over the long term affects mitochondrial homeostasis, favoring the generation of ROS, modifying the balance between fission and fusion, altering the function of the respiratory chain, decreasing the mitochondrial membrane potential and triggering apoptosis. Thus, in a study conducted by Carneiro et al., exposure of dopaminergic neurons to therapeutic concentrations of MPH or amphetamine did not induce changes in the mitochondrial membrane potential. However, exposure to pro-oxidant substances induced changes and generated ROS, which were partially prevented by MPH or amphetamine, suggesting a potential protective effect of MPH in the presence of toxic agents [127]. Studies in rodents show that ROS production is dose-dependent and is correlated with the influence on mitochondrial activity. Thus, in adult rats, acute or chronic administration has been shown to inhibit complexes I, II, III and IV in the hippocampus, prefrontal cortex, striatum and cortex, but not in the cerebellum [34]. Another study in young rats suggests that the activity of mitochondrial complexes II and IV is increased, so that the effects are not only dose-dependent, but also age-dependent [128]. In a study by Rieder et al. in young rats administered MPH (2 mg/kg), the balance between DRP1 and MFN2 was altered, with favoring fission and decreasing mitochondrial fusion in males. In females, NRF1 expression decreased, and Parkin increased. These data suggest that early exposure negatively alters the balance between mitochondrial fission and fusion, and is sex-dependent [129]. Exposure to high doses (10–20 mg/kg) promotes lipid peroxidation, increases GSSG levels and decreases GSH, increases IL-1β and TNF-α, decreases SOD, GPx and GR activity in the hippocampus and cortex [38]. A recent study suggests that exposure to MPH generates oxidative stress and mitochondrial alteration in the retina through NOX2/PI3K/AKT/DRP1. Thus, mitochondrial fission, the alteration of mitochondrial membrane potential and the shift in metabolism to a glycolytic one were favored. In inflammation, the effects of MPH were beneficial, favoring antioxidant mechanisms [130]. Another recent study confirms the positive effects of MPH in the presence of other aggressors, with increased SOD and GPx activity in the heart, decreased MDA and positive modification of mitochondrial dynamics induced by tramadol administration by modifying the expression of DRP1 and PINK-1, in the heart [131].

## 7. Nutritional and Gut–Brain Axis Influences on Redox Balance

Recently, there has been increasing interest and importance in nutrition and the microbiome in modulating ADHD symptoms. Although MPH administration improves symptoms in the short term, its efficacy may be low, although methylphenidate may bring benefits from other points of view (reduces the risk of substance abuse, criminal convictions, suicidal behavior). For this reason, an approach focused on factors related to lifestyle, diet and nutrition has been proposed, suggesting that nutritional deficiencies intervene in behavior modification. In the emerging context of interdisciplinary research, the interaction between diet, microbiome and the functioning of the gut–brain axis opens interesting perspectives for understanding and addressing ADHD. The gut–brain axis involves several intrinsic pathways, one of which is neurochemical, on which the microbiome influences the synthesis of dopamine, serotonin and GABA, another pathway is immune, demonstrated by the fact that alteration of the microbiome increases intestinal permeability with an increase in proinflammatory cytokines, and another pathway is metabolic, represented by short-chain fatty acids and antioxidant systems [132].

Also, a systematic review states the existence of differences in the composition of the microbiome in patients with ADHD, with a decrease in beneficial genera and an increase in genera associated with inflammation, at the same time dysbiosis affecting the metabolism of dopamine and serotonin precursors [133,134,135]. Short-chain fatty acids also intervene in the synthesis of monoamines by influencing the enzymes involved [135]. The amino acids that are precursors of monoamines are also produced by the microbiota, which strengthens the connection between the gut and the brain. Serotonin and gamma-aminobutyric acid are mediators involved in ADHD, so the microbiome can influence the absorption and secretion of these neurotransmitters [136,137,138]. Thus, a change in the microbiome or dysbiosis causes an increase in the species Actinobacteria, Bifidobacterium, Odoribacterium, Bacteroides, and a decrease in Faecalibacterium in children and adolescents with ADHD [133]. A diet dominated by processed foods, with a high content of carbohydrates, unhealthy fats, is associated with an increased incidence of ADHD symptoms. However, a sufficient intake of omega-3, iron, zinc and B vitamins has a protective effect [139]. After the breakdown of fibers, short-chain fatty acids appear, which represent an energy substrate for mitochondria. Short-chain fatty acids also activate PGC-1α and PPAR-α, which influence mitochondrial dynamics and increase antioxidant activity and promote anti-inflammatory effects [140,141]. Increased ROS levels and impaired mitochondrial activity promote the generation of more ROS, which oxidize polyunsaturated fatty acids, activate microglia, and release proinflammatory cytokines. One study suggests that IL-16 and IL-13 levels in children with ADHD are increased and correlate with ADHD symptoms [142,143]. Due to its high content of vitamins, fiber, and antioxidants, the Mediterranean diet is proposed to reduce inflammation and increase the intake of polyphenols and fermentable fibers [138,144,145,146]. A diet that includes polyphenols has the possibility of modulating oxidative stress by activating Nrf2, with the stimulation of antioxidant enzymes, having neuroprotective potential [145,146].

Adopting a Mediterranean diet rich in flavonoids reduces oxidative stress and symptoms of hyperactivity [147,148], while in animal models, polysaccharide extracts restore the microbiome, reducing permeability and inflammation [149]. The inclusion of omega-3 fatty acids in children with ADHD has resulted in a decrease in inflammatory markers, oxidative stress and hyperactivity symptoms Effect of n-3 supplementation on hyperactivity, oxidative stress and inflammatory mediators in children with attention-deficit-hyperactivity disorder, as more short-chain fatty acids and bacterial fermentation products are generated [150,151]. These effects are due to positive effects on microbiome composition, decreased intestinal permeability, endotoxemia and inflammation, thus suggesting the need to include omega-3 fatty acids in the diet. Also, for antioxidants to have good bioavailability, a microbiome is needed to ensure this. Antioxidant supplements increase intestinal barrier integrity and reduce lipopolysaccharide translocation, decreasing microglial activation and neuronal inflammation. Another proposed diet is the ketogenic diet, which has been applied in an experimental animal study and suggests an improvement in ADHD symptoms by regulating the microbiome [152].

## 8. Forensic Toxicology and Detection in Abuse Contexts

Currently, the consumption and trafficking of substances with potential for abuse is a concern, and analytical methods are continuously used and improved to combat these phenomena. These methods are essential in the rapid and accurate discovery of the compounds in those products, especially since new substances are introduced on the market, which have not been included in any list [7]. The methods often used are gas chromatography (GC), high-performance liquid chromatography (HPLC), mass spectrometry (MS), infrared spectroscopy (IR, FTIR). They allow the detection and determination of the concentrations present in the body of users, providing information on the confirmation of consumption and the degree of intoxication. A characteristic of these methods is their sensitivity and specificity, since samples can contain very low concentrations of substance or metabolite, as seen in Table 4.

Saliva and blood are the matrices most often used for the detection of narcotic substances and provide data on acute use and recent consumption, while urine provides data for extended detection, but without establishing the state of intoxication. An LC-MS/MS method developed by Josefsson et al. allows the determination of MPH and ritalinic acid in plasma, blood and saliva. With this method, MPH concentrations were determined 4 times higher in saliva than in blood, and the concentration of ritalinic acid is 25 times lower in saliva [153]. Another method using blood as a matrix is based on enantioselectivity, to establish the ratio between the L and D enantiomers of MPH and ritalinic acid. This method did not observe a post-mortem redistribution of the enantiomers. The method brings advantages due to the differences in potency between the enantiomers. The D enantiomer is the active isomer, while the L enantiomer has a reduced pharmacological activity. Thus, the ratio between the two forms can provide information related to active consumption or the residual presence of the substance in the body. Also, pharmaceutical products contain the racemic mixture, and if only the D-isomer is present in the body, it is possible that it is due to abuse [154]. A matrix that can provide long-term information on abuse is re-presented by hair. The method is advantageous because it is non-invasive, the sample is stable, allows the identification of both MPH and ritalinic acid, so that the presence of the metabolite indicates the consumption of the substance and not a contamination of the matrix, and the ratio between the concentrations of the two substances provides information on the mode of administration. The method is not only useful from the point of view of abusive use, but also for doping in the case of athletes and compliance with treatment [155]. There are also methods that use the brain, in post-mortem studies. The use of this matrix provides information on the mode of distribution of the substance useful in understanding the mechanisms of toxicity, oxidative stress, neuroinflammation, the effect of redistribution of the substance is avoided [156].

**Table 4:** Table of matrices used for determining methylphenidate (MPH) and ritalinic acid (RA) concentrations.

Matrices	Method	Linearity/Sensitivity	References
Hair	LC-MS/MS	Linearity: 1–100 pg/mg LOD: 0.5 pg/mg for MPH, 1 pg/mg for RA LOQ: 1 pg/mg for both analytes	[155]
Hair	LC-MS	Linearity: 0.15–50 ng/mg LOD: 0.05 ng/mg LOQ: 0.15 ng/mg	[156]
Hair	LC-HRMS	Linearity: 1–40 pg/mg LOD: 0.3 pg/mg LOQ: 1 pg/ng	[157]
Blood Plasma Oral fluid	LC-MS/MS	Linearity: 0.2–30 ng/mL for MPH, 10–1500 ng/mL for RA in blood and plasma, and 1–500 ng/mL for MPH and 0.25–125 ng/mL for RA in oral fluid LOQ: 0.2 ng/mL for MPH and 5 ng/mL for RA in blood, 0.1 ng/mL for MPH and 2.5 ng/mL for RA in plasma and 0.1 ng/mL for MPH and 1 ng/mL for RA in oral fluid	[153]
Blood	LC-MS	Linearity: 200–25,000 pg/mL LOQ: 200 pg/ml	[158]
Blood	LC-MS/MS	Linearity: 0.2–500 ng/g LOQ: 0.5 ng/g	[154]
Urine	LC-MS/MS	Linearity: 5–5000 μg/L LOD: 5 μg/L LOQ: 100 μg/L	[159]
Oral fluid	LC-MS/MS	Linearity: 0.5–50 ng/mL LOQ: 0.5 ng/	[160]

LOD—limit of detection; LOQ—limit of quantification.

## 9. Risks of Binge/Crash

Abusive use of stimulants such as MPH, amphetamines, or cocaine often follows a compulsive pattern characterized by periods of binge consumption followed by crashes with insufficient recovery time. This alternating cycle of excessive stimulation and exhaustion creates a self-perpetuating state of chronic oxidative stress. During the binge phase, large amounts of ROS are generated as a result of catecholamine metabolism, which alters the activity of antioxidant systems in the prefrontal regions and limbic system. Animal studies with amphetamines or cocaine confirm that repeated binges cause a persistent decrease in the GSH/GSSG ratio and in the activities of SOD, glutathione peroxidase, and glutathione reductase [161,162,163]. Crash symptoms include severe fatigue, apathy, depression, anxiety, and cognitive dysfunction. The binge/crash pattern forms a vicious cycle, with episodes increasing in intensity and duration, predisposing individuals to psychosis, chronic anxiety, and cognitive deficits. The lack of redox recovery between episodes leads the body into a chronic state of stress.

Addiction is a complex disorder involving profound neurological remodeling. Increasing evidence suggests that one reason for this vulnerability is the relationship between oxidative stress and chronic neuroinflammation, which reinforces relapse mechanisms. In the early stages of use, increased DA and glutamate release triggers ROS production and the release of proinflammatory cytokines. This creates a circuit where oxidative stress activates microglia and astroglia to release interleukins (IL-1β, TNF-α), intensifying the inflammatory response and disrupting dopaminergic synapses. Preclinical studies show strong correlations between oxidative stress, neuroinflammation, and relapse. A study on rats with chronic alcohol consumption revealed that both oxidative and inflammatory markers remain elevated long after the abstinence period [164,165,166].

The benefit-risk assessment of MPH is difficult because study results vary depending on factors such as study design, patient age, dosage, comorbidities, and follow-up. One such example is cardiovascular risk. A study by Cooper W.O. et al. in children and adolescents found no association between current ADHD medication treatment (MPH, dexmethylphenidate, dextroamphetamine, amphetamines, atomoxetine, pemoline) and serious cardiovascular events, indicating relative short-term safety [167]. A recent meta-analysis confirmed that there is no overall increased risk of cardiovascular disease but acknowledged that a small risk of tachyarrhythmias or cardiac arrest cannot be completely excluded, especially in women and patients with pre-existing cardiovascular disease [168]. A study in adults also suggested a slightly increased cardiovascular risk in the first six months of treatment, but an increase in risk of more than 20% is unlikely [112]. The discrepancies between these studies stem from differences in age, the period of risk analyzed (start of treatment or maintenance of treatment), and the way in which confounding factors (cardiovascular history, smoking) were controlled.

A study by Man et al. reported a higher number of suicide attempts immediately before and shortly after starting MPH treatment, but the authors noted that this may be due to patients having severe symptoms, which predispose them to such behavior [169]. Another study suggests that ADHD medications are associated with significant reductions in the risk of suicidal behavior, problematic substance use, road traffic accidents, and crime, supporting the idea of a net benefit in real-world, well-controlled settings [170]. A meta-analysis based on observational data showed that not only does stimulant treatment in childhood not increase the risk of later substance abuse, but it may even have a protective effect [171].

In addition to these clinical aspects, research is also exploring other areas, such as interaction with the gut microbiota. Preliminary data suggest that psychostimulant drugs may modulate short-chain fatty acids and microbiota composition, but without being able to clearly distinguish the effect of the drug from that of diet or ADHD itself [172,173]. Interestingly, on the pharmacokinetic side, there is evidence that MPH degradation in the gut may be caused by pH-dependent hydrolysis rather than bacterial metabolism, suggesting that some of the variability in drug efficacy may be chemical, not microbial, in nature, an area that requires more clinical studies [174].

## 10. Conclusions

Although it is effective in the treatment of ADHD and narcolepsy, the diversion of this substance from clinical use may lead to alterations in redox balance and behavior, as suggested by various experimental animal studies, with potential neurotoxic risks associated with long-term abusive use. Longitudinal studies are imperative to answer questions related to the effects on oxidative stress and mitochondrial homeostasis, as most data come from preclinical studies. In this context, interest in antioxidants is increasing (vitamin E, coenzyme Q10, N-acetylcysteine, resveratrol, polyphenols, flavonoids), the aim being to reduce oxidative effects on the brain, liver, heart and kidneys. Associated with antioxidants, mitochondrial therapies offer another direction. In other clinical contexts, compounds that influence mitochondrial activity have been tested (NAD+ donors, nicotinamide riboside, for the maintenance of mitochondrial complex activity, PGC-1α activators, mitochondrial biogenesis regulator, targeted antioxidants, MitoQ, SkQ1, melatonin). The aim includes not only the detection of abusive use and the recording of adverse effects, but also the development of protective strategies before toxicity becomes irreversible.

## Figures and Tables

**Table 1:** Doses and purpose of methylphenidate use.

Purpose of Use	Dose	Frequency	Route of Administration	References
**Therapeutic for children/adults**	0.2–0.7 mg/kg 20–30 mg/kg	2–3/day IR 1/day ER	Oral	[16,17,18]
**Therapeutic**	10, 15, 20, 30 mg	1/9 h	Transdermal patch	[19]
**Abuse**	700 mg	Occasionally (for 3 days)	Intranasal	[20]
**Abuse**	10, 20, 30 mg	Repeated	Intranasal	[21]
**Abuse**	40–1000 mg	Repeated	Intravenous	[22]

IR—immediate release; ER—extended release.

**Table 2:** Effects of methylphenidate according to dose.

Route of Administration	Dose	Duration	Behavioral Assay	Effects	References
**Drinking water**	20, 30, 60 mg/kg/day Dual doses: 4/10, 20/30, 30/60 mg/kg/day	11 weeks	Open Field, Anxiety, Locomotor activity	Increase anxiolytic behavior, decreased exploratory behavior	[43]
**Oral**	3 mg/kg	18 days	Radial Arm Maze	Improved spatial learning and memory	[44]
**Oral**	10 mg/kg	Acute	Object Recognition	Impaired memory	[45]
**Oral**	2, 3, 5 mg/kg	11/21 days	Open Field, Object Recognition	Impaired recognition memory for rats treated with 3 or 5 mg/kg for 21 days	[46]
**Oral**	2, 5 mg/kg	Twice daily for 7 weeks	Open Field Novel Object Recognition Contextual fear	Does not produce cognitive impairments	[47]
**Oral**	2 mg/kg	13 days	Elevated Plus Maze, Social interaction, Open Field, Object in place recognition	The environment modifies methylphenidate effects	[48]
**Oral**	1.5, 5 mg/kg	28 days	Open Field	High doses induce anxiety behavior	[49]
**Oral**	20 mg/kg	21 days	Rotarod	Decreased coordination	[50]
**Subcutaneous**	0.6, 2.5, 10, 40 mg/kg	Acute	Open Field	The 2.5, 10, and 40 mg/kg increased locomotor activity	[51]
**Subcutaneous**	1, 5 mg/kg	Prior aversive conditioning	Aversive conditioning	Increased learning	[52]
**Intraperitoneal**	2 mg/kg	30 days	Open Field, Morris Water Maze	Impaired spatial memory and working memory	[53]
**Intraperitoneal**	5 mg/kg	Twice daily (for chronic treatment) for 7 days A single dose of 5 mg/kg	Object exploration in Circular Open Field	Alters recognition memory, no effect on locomotor activity	[54]
**Intraperitoneal**	2 mg/kg	28 days	Inhibitory avoidance test, Continuous multiple inhibitory avoidance	The age and time of treatment can alter learning and memory	[55]
**Intraperitoneal**	2 mg/kg	Twice daily for 15 days	Sucrose Preference test, Elevated Plus Maze, Forced Swimming, Open Field	Decreases sucrose preference, causes anxiety, and stress	[56]
**Intraperitoneal**	2 mg/kg	Twice daily for 16 days	Place Conditioning, Forced Swimming, Open Field	High doses of cocaine given appeared less rewarding, causes depressive effects, reduced habituation	[57]
**Intraperitoneal**	2 mg/kg	Twice daily for 14 days	Fear conditioning	No effects during the fear acquisition, increased anxiety-like behavior	[58]
**Intraperitoneal**	2 mg/kg	Twice daily for 16 days	Play behavior, Sucrose Preference, Novel Environment, Elevated Plus Maze, Social interaction, Sexual behavior, Forced Swimming	Decreased response to sucrose, novel environment, and sexual behavior. Increased anxiety-like behavior	[59]
**Intraperitoneal**	10 mg/kg	21 days	Open Field, Forced Swimming, Elevated Plus Maze, Tail suspension, Morris Water Maze	Increased depression and anxiety behavior. Decreased locomotor activity	[60]
**Intraperitoneal**	2 mg/kg	21 days	Sucrose Preference	Increased anxiety behavior	[61]
**Intraperitoneal**	10 mg/kg	5 days	Morris Water Maze, Forced Swimming, Open Field	Antidepressant effect and increased anxiety behavior	[62]
**Intraperitoneal**	5, 50 mg/kg	15 days for 5 mg/kg Acute for 50 mg/kg	Inhibitory avoidance, Object recognition	Acute treatment with 5 mg/kg improved memory while acute treatment with 50 mg/kg decreased memory in avoidance test. High doses impaired memory recognition	[63]
**In food**	1 mg/kg	17 weeks	Morris Water Maze	No change in visual learning	[64]

**Table 3:** Brief table with comparative toxicities on retina, liver, kidneys, and heart.

Organ	Toxic Effects	Mechanism	References
Retina	Decreased photoreceptor viability (661 W cells), caspase activation, ROS (reactive oxygen species) accumulation, MDA (malondialdehyde) increase, dysregulated autophagy	Oxidative stress, apoptosis, autophagy	[95]
Liver	Increased liver enzymes, liver failure	Metabolic disorders, enzymatic adaptation, oxidative stress	[113,114,115,116,117]
Kidneys	Necrosis, inflammation, cellular infiltrate, decreased renal corpuscle volume and Bowman’s space, increased BUN (blood urea nitrogen) and creatinine; partially reversible with ATP	Inflammation, apoptosis, dysregulated autophagy, oxidative stress, NF-κB activation	[118,119]
Heart	Interstitial edema, vascular congestion, fibrinous material; oxidative stress; at very high doses, minor changes in QT and blood pressure; ischemia–reperfusionischemia-reperfusion does not worsen lesions	Oxidative stress, inflammation, hemodynamic disturbances at high doses	[110,120,121]

## Data Availability

No new data were created or analyzed in this study.

## Abbreviations

The following abbreviations are used in this manuscript:

ADHD	Attention deficit hyperactivity disorder
DA	Dopamine
NA	Norepinephrine
5-HT	Serotonin
DAT	Dopamine transporter
NET	Norepinephrine transporter
VMAT2	Vesicular monoamine transporter 2
CES1	carboxylesterase 1
ROS	Reactive oxygen species
IR	Immediate release
iNOS	Inducible nitric oxide synthase
ER	Extended release
8-OHdG	8-hydroxy-2’-deoxyguanosine
MDA	Malondialdehyde
TBARS	Thiobarbituric acid reactive substances
SOD	Superoxide dismutase
CAT	Catalase
GPx	Glutathione peroxidase
MAO-B	Monoamine oxidase type B
NMDA	N-methyl-D-aspartate
AGEs	Advanced glycation end products
RAGE	Receptors of advanced glycation end products
MDMA	Methylenedioxymethamphetamine
CK	Creatine-kinase
GSH/GSSG	Reduced glutathione/oxidized glutathione ratio
NF-kB	Nuclear factor kappa B
NRF1	Nuclear respiratory factor 1
Nrf2	Nuclear factor erythroid 2-related factor 2
BUN	Blood urea nitrogen
NOX2	NADPH oxidase 2
PI3K	Phosphoinositide 3-kinase
AKT	Protein kinase B
DRP1	Dynamin-related protein1
MFN2	Mitofusin 2
PINK1	PTEN-induced kinase 1
